# Replacing 2 Gy Per Fraction Equivalent Dose with Fractionation-Specific Biological Equivalent Dose for Normal Tissues

**DOI:** 10.3390/ijms252312891

**Published:** 2024-11-30

**Authors:** Wei Luo, William St Clair

**Affiliations:** Department of Radiation Medicine, University of Kentucky, Lexington, KY 40536, USA; stclair@email.uky.edu

**Keywords:** 2 Gy per fraction equivalent dose (EQD_2_), fractionation-specific biological equivalent dose (FEQD(n)), biological effective dose (BED), linear-quadratic equation (LQ)

## Abstract

The 2 Gy per fraction equivalent dose (EQD_2_) is an important quantity used in determining equivalent prescription doses for different fractionation regimens and evaluating different fractionation regimens, but it does not match its definition when it is used for normal tissues. We propose to use the fractionation-specific biological equivalent dose to determine normal tissue dose constraints for different fractionation regimens. The concept of the biological equivalent dose is defined based on the linear-quadratic equation. The EQD_2_ is derived based on the biological effective dose (BED), mimicking the prescription dose of a standard fractionation regimen with a fractional dose of 2 Gy and a fixed number of fractions. The FEQD(n) is also defined based on the BED as a function of the number of fractions, n, which is determined by the dose prescription. The FEQD(n) mimics any fractionation regimens with any fractional doses and numbers of fractionations. A given dose constraint can have different BED values and EQD_2_ values for different fractionation regimens. The number of fractions for a given 2 Gy per fraction regimen derived from the EQD_2_ for the target dose is different from that for the normal tissues. The value of the EQD2 derived for the target represents the total dose for the target for the 2 Gy fractional dose regimen, but the EQD_2_ value derived for the normal tissues does not represent the total dose for the normal tissue for the same fractionation regimen. The fractionation-specific biological equivalent dose (FEQD(n)) for both target and normal tissues has the same number of fractions for any fractionation regimen, and represents the total dose for either the target or the normal tissue. Based on the clinical outcomes, the FEQD(n) curves for the brainstem, spinal cord, rectum, and lung were derived and can be directly used as dose constraints for various fractionation regimens in clinical practice. The EQD_2_ does not match its definition and is not realistic when describing the biological equivalent dose for normal tissues. It is also not practical when used in determining tolerance doses or dose constraints. Instead, the FEQD(n) can be used to determine or convert the normal tissue dose constraints for any fractionation regimens in a realistic and practical manner. Using the FEQD(n), the dose constraints as a function of the number of fractions for the brainstem, spinal cord, rectum, and lung, which correspond to the given toxicity rates, were derived and can be directly used in clinical practice.

## 1. Introduction

Radiation can cure cancer but can also injure normal tissues. In radiation therapy, the dose to normal tissues should be limited to within their tolerance to protect them. Certain tolerance doses for relevant tissues or organs have been proposed and used as dose constraints in clinical practice [1,2,3]. The tolerance dose for each organ can be derived from clinical outcomes for specific treatment techniques and fractionation regimens. However, collecting and analyzing clinical outcome data are long-term tasks and limited to treatment techniques, treatment sites, and fractionation regimens. It is not practical to test tolerance doses for every patient, especially when new fractionation regimens are introduced. An alternative is to derive the tolerance doses for a new fractionation regimen from a known regimen based on the equivalent biological effect. This biological effect may be correlated to clinical outcomes that include tumor control and normal tissue toxicities or complications. If a relationship between the cell survival fraction (S) and radiation dose (D) is established, a tolerance dose corresponding to a value of S can be determined. The relationship between S and D can be derived from experimental data and clinical results, and plotted as the S-D curve.

To compare different fractionation regimens, F. Ellis first proposed a quantity termed the Nominal Standard Dose (NSD) based on the survival curves (S-D curve) for mammalian cells such as Hela cells and human skin cells [4]. It was found that the values of the NSD are similar for different fractionation regimens with different tolerance doses. Thus, it was suggested that the NSD be used to compare different fractionation regimens in terms of normal tissue tolerance. The NSD was further generalized as the Cumulative Radiation Effect (CRE) [5]. Another factor called the Time, Dose, and Fractionation (TDF) factor was introduced to simplify the use of the NSD [6]. However, the NSD was found to be inaccurate in estimating the tolerance dose for late reactions [7]. J. Fowler pointed out that the NSD overestimated the tolerance dose for late reactions and underestimated it for early reactions [8]. Meanwhile, other biological models based on the S-D curves have been introduced to predict normal tissue tolerance doses. Since the linear-quadratic (LQ) model can fit the S-D curve very well [7,8,9,10], it has been widely accepted and used in clinical practice. While more complicated models were proposed to better fit the S-D curves for very large fractional doses [11,12,13,14], they are not clinically used. The analysis in this study was based on the LQ model.

Based on the LQ model, the biological effective dose (BED) has been defined as a function of S and D for an S-D curve [8,12,13,14,15,16,17,18,19,20]. The BED is proportional to the logarithm of S and it can be used to describe the effectiveness of radiation. Another important concept, the biological equivalent dose (EQD), can be defined based on the BED. If two cell survival curves for different fractionation regimens reach the same survival fraction after they have received two different doses, the two different doses are biologically equivalent since they have killed the same number of cells. Biological equivalent doses can be converted into one another. As the fractional dose of 2 Gy has been used in clinical practice for a long time and has rich clinical outcome data, the 2 Gy fractional dose regimen is considered as a standard fractionation regimen and can be used as a reference to assess other fractionation regimens. Thus, other fractionation regimens are usually converted into the 2 Gy regimen for outcome assessment. If a given fractionation regimen has the total dose D after being converted into a 2 Gy regimen with the total dose D_2_, the D is equivalent to D_2_, and D_2_ is labeled as the EQD_2_ [8,15,16,17,18,19,20], where the subscript “2” refers to “2 Gy”. In general, the value of D for a given fractionation regimen is different from the value of its corresponding EQD_2_. It is a common practice to convert the target dose and normal tissue tolerance doses for various fractionation regimens into the EQD_2_ for clinical outcome assessment.

Using the EQD_2_ to convert a prescription dose or target dose into 2 Gy fractions is completely acceptable since 2 Gy is a standard and actual prescription dose; however, it would cause confusion to use the EQD_2_ for normal tissue dose constraints. First of all, the actual dose for critical structures is not usually a fixed 2 Gy per fraction. Thus, it is not realistic to use the EQD_2_ to mimic the 2 Gy fractionation regimen for normal tissues. Secondly, dose constraints or tolerance doses used in treatment plans are not described in EQD_2_ but in actual dose. Even if the EQD_2_ is given, it has to be converted into the actual dose, which is not practical in treatment planning. In this study, we defined the fractionation-specific equivalent dose (FEQD(n), where n is the number of fractions) to determine equivalent doses for various fractionation regimens. We used it to calculate the fractionation-specific dose constraints or tolerance doses for the organs at risk (OARs), including the brainstem, spinal cord, lung, and rectum.

## 2. Results

### 2.1. Variation of BED with Number of Fractionations for the Same Normal Tissue Dose

According to Equation (4), the BED is a function of D or d and n and varies with D or n. The following calculations are based on standard head and neck IMRT with a prescribed fractional dose of 2 Gy. The EQD_2_ is calculated by Equation (12). The three prescribed doses, 60 Gy, 66 Gy, and 70 Gy, give three different BED values and different numbers of fractions (n = 30, 33, 35). A dose limit of 54 Gy (D_max_) has been widely used as a generic maximum dose constraint for the brainstem in conventional radiation therapy regardless of the different numbers of fractions. [3] However, it results in three BED values with differences of up to 6.4%, indicating that the same dose constraint should not be used for different fractionation regimens. Meanwhile, the calculated EQD_2_ values cannot be directly used as dose constraints in treatment plans and are not needed in this case. The results are shown in Table 1. The α/β of 10 Gy was used for the tumor target and 2.5 Gy for the brainstem.

### 2.2. Variation of FEQD(n)/FEQD_2_(n) with Number of Fractions for the Same BED

The brainstem maximum dose limit varies with n and the values. For the three fractionation regimens given above, the dose constraints can be determined using the FEQD_2_(n). The FEQD(n)/FEQD_2_(n) is calculated by Equations (10)–(17). Corresponding to the three different BED values of 92.88 Gy, 89.35 Gy, and 87.33 Gy, the FEQD_2_ varies with n for each BED value, as shown in Table 2. The EQD_2_ is not the actual dose received by the brainstem, while the FEQD_2_(n) is the actual dose and can differ by up to 11.3% from the EQD_2_. Notably, given the BED values, the FEQD_2_(n) values can be derived as dose constraints without a need for the EQD_2_.

### 2.3. Determination of Dose Constraints Using FEQD(n)/FEQD_2_(n) Based on Clinical Outcomes

In this study, the FEQD(n) formulism (Equations (10)–(17)) was used to derive tolerant doses or dose constraints for critical structures from original clinical outcome data. The original dose constraint for a relevant organ at risk (OAR) was derived from the published clinical data, which included the original prescribed dose, number of fractions, maximum or mean dose, and the corresponding toxicity rate. Then, the BED was calculated for both the target dose and the dose constraint for the relevant OAR. Based on the target BED, the original fractionation regimen was converted into the standard 2 Gy per fraction regimen with a new number of fractions, n, and the corresponding FEQD_2_(n) for the OAR was calculated based on n and the OAR’s BED, which corresponds to a toxicity rate. The FEQD_2_(n) was compared with the EQD_2_. Finally, the FEQD(n) values as the dose constraints for the brainstem, spinal cord, rectum, and lung were derived and plotted against the number of fractions ranging from 1 to 45. Those values correspond to the toxicity rates determined by the clinical outcomes.

#### 2.3.1. Brainstem

To determine the brainstem’s tolerance dose, we used the brainstem complication data reported in three studies. The first report was published by Debus et al. [21]. A total of 367 patients with chordomas and low-grade chondrosarcomas of the base of the skull were treated with high-dose photon and proton irradiation and 17 patients experienced brainstem toxicity, which was 4.6% of the total patients. The prescription doses ranged from 66.6 Gy to 77.4 Gy with 37 to 43 fractions of 1.8 Gy for the 17 patients. The tolerance dose for the brainstem was set to 60 Gy, but the maximum doses actually received by the brainstem among those patients ranged from 60 Gy to 68.6 Gy. On average, the prescribed dose was 70.3 Gy in 39 fractions. The averaged brainstem maximum dose was 64.3 Gy in 39 fractions.

The BED for the averaged target dose was obtained to be 83.00 Gy, and the corresponding EQD_2_ for the tumor was 69.15 Gy with a number of fractions of 35. The BED for the brainstem maximum dose was 106.9 Gy, which corresponded to the brainstem toxicity rate of 4.6%. Using the brainstem BED, it was found that the biological equivalent dose (FEQD_2_) of the averaged brainstem maximum in 35 fractions was 62.2 Gy. It was also found that the EQD_2_ for the averaged brainstem maximum was 59.4 Gy. The difference between the FEQD_2_ and EQD_2_ was 2.8 Gy, or 4.7%. The results are listed in Table 3.

The second report included a total of 40 benign meningioma patients treated with IMRT [22]. The prescribed dose ranged from 40 to 56 Gy and the fractional dose ranged from 1.71 to 2 Gy. Only one patient experienced brainstem injury, i.e., a 2.5% injury rate, and the corresponding brainstem BED was 99.76 Gy. This patient was treated to a prescribed dose of 50.4 Gy in 28 fractions. The maximum dose to the brainstem was 55.6 Gy. The BED calculation for meningioma uses an average α/β of 3.5 Gy according to Dhere et al. [23]. The difference between the FEQD_2_ and EQD_2_ was −2.44 Gy, or −4.4%.

**Table 3:** Comparisons between FEQD_2_ and EQD_2_ derived from real cases for the brainstem.

Structure	Reference	BED (Gy)	D (Gy)	d (Gy)	n	α/β (Gy)	Toxicity Rate
Target	D = Prescribed dose						
	Debus [21]	83.00	70.30	1.80	39	10	
	Uy [22]	76.32	50.40	1.80	28	3.5	
	Schoenfeld * [24]	63.72	54.00	1.80	30	10	
		20.70	18.00	1.50	12	10	
	D = FEQD_2_						
	Debus	83.30	69.15	2.00	35	10	
	Uy	76.32	48.57	2.00	24	3.5	
	Schoenfeld	84.42	70.35	2.00	35	10	
Brainstem	D = D_max_						
	Debus	106.9	64.30	1.70	39	2.5	4.6%
	Uy	99.76	55.60	1.99	28	2.5	2.5%
	Schoenfeld	89.40	55.00	1.56	35	2.5	0.0%
	D = FEQD_2_						
	Debus	106.9	62.20	1.80	35	2.5	4.6%
	Uy	99.76	52.98	2.21	24	2.5	2.5%
	Schoenfeld	89.40	55.00	1.56	35	2.5	0.0%
	D = EQD_2_						
	Debus	106.9	59.40	2.00	30	2.5	4.6%
	Uy	99.76	55.42	2.00	28	2.5	2.5%
	Schoenfeld	89.40	49.67	2.00	25	2.5	0.0%

Note: D represents prescribed dose for target and maximum dose for the brainstem, d is the fractional dose, n is the number of fractions. * Two prescriptions were used for the same treatment.

**Figure 1:**
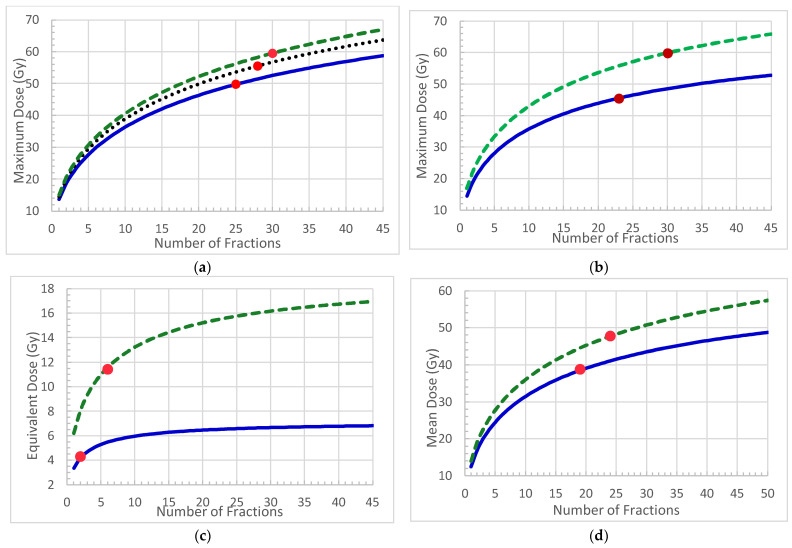
FEQD(n) curves for the critical structures. (**a**) Brainstem mean dose curves with toxicity rates of 4.9% (green dashed line), 2.5% (black dotted line), and 0.0% (blue line); (**b**) spinal cord maximum dose curves with myelitis rates of 2.5% (green dashed line) and 0.4% (blue line); (**c**) rectum mean dose curves with bleeding rates of 7.5% (green dashed line) and 3.0% (blue line); (**d**) lung mean dose curves with pneumonitis rates of 13.5% (green dashed line) and 1.6% (blue line). The red dots on the curves are the EQD_2_ for the same BED of the corresponding curves.

The third brainstem study results we used were the brainstem complication data reported by Schoenfeld et al. [24]. In 100 consecutive patients treated with IMRT for squamous cell, no brainstem damage was found, or 0%. The patients were treated using two schemes: a 54 Gy prescribed dose in 30 fractions for the primary treatment and 18 Gy in 12 fractions for the boost, which resulted in an EQD_2_ of 70.35 Gy in 35 fractions. Correspondingly, the biological equivalent maximum dose to the brainstem was 55 Gy (FEQD_2_) in 35 fractions and its EQD_2_ was 49.67 Gy, which corresponded to an 89.40 Gy BED. The difference between the FEQD_2_ and EQD_2_ was 5.33 Gy, or 10.7%. The results from the analysis of all three data sets are summarized in Table 3. Three FEQD(n) curves corresponding to the three BEDs and toxicity rates were also calculated and are plotted in Figure 1a.

#### 2.3.2. Spinal Cord

In radiation therapy, dose constraints for the spinal cord are critical for preventing cord injury. Marcu et al. reported that 2 out of 471 patients (0.4%) experienced myelitis after radiation treatment for malignancies of the upper respiratory tract [25]. Wara et al. reported the results for 172 lung and head and neck patients who received radiation therapy. A total of nine patients (5.2%) suffered spinal cord injury [26]. We used 3.9 Gy as the α/β for the spinal cord BED calculation in this study [27]. The results of the BED and equivalent dose calculations are summarized in Table 4. Two FEQD(n) curves corresponding to the two BEDs and toxicity rates were calculated and are plotted in Figure 1b.

#### 2.3.3. Rectum

Fonteynea et al. [28] reported the clinical outcomes for 241 prostate IMRT patients. The median follow-up was 42 months. Out of the 241 patients, 15 developed Grade 2 red blood bleeding and 3 developed Grade 3 red blood loss, making a total of 18 patients, or 7.5. We used the mean rectum dose for analysis. The average mean rectum dose was 54 Gy for 38 fractions, and the corresponding BED was 79.4 Gy. Al-Mamgani et al. [29] published results for 78 prostate patients, including 41 patients treated with IMRT. They reported a 3% 5-year Grade 2 or higher rectal bleeding rate. The rectum mean dose was 46.3 Gy for 39 fractions and the BED was 64.6 Gy. The results for the BED and EQD for both studies are presented in Table 5. Also, two FEQD(n) curves corresponding to the two BEDs and toxicity rates were calculated and are plotted in Figure 1c.

#### 2.3.4. Lung

Radiation-induced pneumonitis is a concern in lung radiation treatment. The mean lung dose (MLD) was considered to be correlated to pneumonitis. Wang et al. analyzed clinical outcomes data from 223 NSCLC patients treated with concurrent chemotherapy and three-dimensional conformal radiation therapy (3D-CRT) [30]. The 1-year actuarial incidence of Grade ≥ 3 radiation-induced pneumonitis in patients with MLD ≤ 16.5 Gy was found to be 13.5%. Baker et al. included 263 lung SBRT patients in their report and found that 3 patients developed Grade 3 pneumonitis, accounting for 1.6% incidence corresponding to the MLD of 5.3 Gy [31]. The corresponding BED and equivalent doses are listed and compared in Table 6. Again, two FEQD(n) curves corresponding to the two BEDs and toxicity rates were calculated and are plotted in Figure 1d.

#### 2.3.5. A Comparison of Tolerance Doses

The FEQD(n) curves above can be compared with those tolerance doses widely used in clinic. Here, we took several dose points on the curves for the brainstem and spinal cord for SBRT in 1, 3, and 5 fractions. Rephrased: “The dose points described by the FEQD(n) were compared to the dose constraints suggested by Emami, D, and used in clinic for both conventional and SBRT fractionations [3]. The toxicity rates corresponding to FEQD and D were also compared. The tolerance doses and toxicity rates agreed very well. For the brainstem, D = 15 Gy, 23 Gy, 31 Gy, and 64 Gy, compared to FEQD = 15.1 Gy, 24.8 Gy, 30.8 Gy, and 63.9 Gy, n = 1, 3, 5, and 38. The toxicity rates were calculated to be 5% by Emami and 4.9% by this study. For the spinal cord, D = 14 Gy, 23 Gy, 30 Gy, and 50 Gy, compared to FEQD = 14.5 Gy, 23.1 Gy, 28.2 Gy, and 50 Gy, n = 1, 3, 5, and 34. The toxicity rates were calculated to be 1% by Emami and 0.4% by this study.

## 3. Discussion

Effectively limiting the dose to normal tissues to within their tolerance is critical for protecting the critical structures. Since the clinical data for tolerance doses are very limited in clinic, it is difficult to define all dose constraints based on clinical outcomes. As an alternative, the dose constraints for new treatment schemes and new fractionation regimens can be derived as the equivalent tolerance doses from the known tolerance doses. The EQD_2_ has thus been introduced to determine equivalent normal tissue dose constraints. However, conceptual issues arise when the EQD_2_ is used for normal tissues. By definition, the EQD_2_ is a total dose with a fractional dose of 2 Gy mimicking the standard fractionation scheme. Therefore, it should contain the fractional dose (2 Gy) and the total number of fractions. This is the case for a tumor target but not for normal tissues. In most cases, the fractional dose for a normal tissue is not 2 Gy. Moreover, the number of fractions calculated using the normal tissue EQD_2_ is not equal to the actual number of fractions determined by the prescribed target dose, as shown in this study. Thus, the EQD_2_ does not match its definition when it is used for normal tissues. Consequentially, the meaning of the EQD_2_ has been changed for normal tissues and it is not treated as a real dose quantity but an abstract variable with no actual fractional dose and no actual number of fractions for normal tissues.

On the other hand, the FEQD(n) is a more realistic quantity. The FEQD(n) is fractionation dependent and the number of fractions is determined by the prescription or target dose even when it is used for normal tissues. For a 2 Gy per fraction regimen determined by the target dose, the FEQD_2_(n) for the normal tissues has the same number of fractions as the FEQD_2_(n) for the target. In this case, the subscript “2” refers to the fractional target dose but the fractional dose corresponding to the normal tissue is not usually 2 Gy, which is different from the EQD_2_ when used for normal tissues. Also, the FEQD(n), including FEQD_2_(n) derived for a given treatment plan from the known tolerance doses, can be directly used as the dose constraints in this plan but EQD_2_ cannot. Therefore, we recommend using the FEQD(n) including FEQD_2_(n) to represent the biological equivalent dose constraints for normal structures.

Although the EQD_2_ is an abstract variable, it is a function of the BED and thus related to the biological effect. If two fractionation regimens had the same EQD_2_, they would lead to the same biological effect or biological effectiveness and clinical outcomes. The EQD_2_ thus can be used to compare biological effectiveness between different fractionation regimens. This may be the only reason for using the EQD_2_ for normal tissues. However, we already have a better quantity (BED) to compare biological effectiveness. The BED is directly correlated with the cell survival curve and clinical outcomes, while the EQD_2_ is dependent on the BED. Therefore, if the BED is used, the EQD_2_ is not needed. If the EQD_2_ is used to calculate normal tissue dose constraints, three quantities should be calculated. The first one is the BED for the existing normal tissue tolerance dose. The second one is the EQD_2_ corresponding to the BED. The third quantity is the equivalent tolerance dose for a given fractionation regimen. On the contrary, only two quantities (the BED and the tolerance dose) should be calculated if the FEQD(n) is used. Therefore, using the FEQD(n) is more practical than the EQD_2_.

In this study, we derived the dose constraints for various fractionation regimens using the FEQD(n) for following four critical structures based on clinical outcomes: the brainstem, spinal cord, rectum, and lung. We analyzed the published clinical outcome data and calculated the BED values that corresponded to the specific toxicity rates. Using the BED, we calculated the FEQD(n)s for a broad range of numbers of fractions (n = 1–45) corresponding to the BED values. The FEQD(n) values for different toxicity rates were plotted against the number of fractions as dose constraints (Figure 1). Using the FEQD(n) curves, we can determine the tolerance dose or dose constraint for a given number of fractions, which can be directly used for treatment planning. Those curves contain more information than the tables currently used in clinical practice [3]. Similarly, the dose constraints for other structures can also be derived using the FEQD(n), but we will leave that for future studies.

The tolerance doses analyzed in this study were limited to the maximum or mean doses that were correlated with the toxicities of the critical structures. This was because these were the only dose values provided in the original clinical data. However, the correlations between dosimetric parameters and normal structure toxicities are complex. For example, rectal bleeding may not just be correlated to the mean dose but other parameters including volumetric parameters such as V_65_, etc. [32]. Other parameters or models such as the equivalent uniform dose (EUD) and normal tissue complication probability (NTCP) model may help improve toxicity prediction [33]. This can be discussed in another study. In this study, we just illustrated how the fractionation-specific dose calculation methodology was used to establish the potential correlations between normal tissue complications and dosimetric parameters based on clinical outcome data. The correlations of toxicities with other parameters can be established using this methodology.

It should be noted that the BED and FEQD(n) calculation in this study was only based on the standard LQ model, Equation (1). We understand that various modifications have been proposed to better fit certain experimental data [11,12,13,14]. However, considering that controversies about those models were raised in the literature and only the LQ model has been widely used in clinical practice [8,34,35,36], those modifications were not considered in this study. Also, for a long treatment course, repopulation may take place and a time factor associated with repopulation may be needed for BED calculation [37]. Considering that the time factor may not be accurate, a time factor was not included in this study. Nevertheless, the BED and FEQD(n) formulism will be modified, including relevant factors such as time, oxygenation, vascular damage, the abscopal effect, and the bystander effect [38,39,40,41,42], when enough supporting experimental and clinical results are available. On the other hand, since all models include certain approximations and assumptions, the BED and FEQD(n) calculations based on either the LQ model or modified models include uncertainties. Consequently, the dose constrains derived from the BED and FEQD(n) should be tested in clinical practice and adjusted if needed based on the corresponding clinical outcomes.

## 4. Method and Materials

### 4.1. Linear-Quadratic (LQ) Equation and BED

The linear-quadratic (LQ) equation is given by

(1) $$S=e^{\left(-\alpha d-\beta d^{2}\right)}$$

where *S* is the cell survival fraction, *d* is the dose for a single fraction, and *α* and *β* are fitting parameters determined by experiments. BED is defined as

(2) $$BED=-\frac{lnS}{\alpha }$$

*BED* is proportional to the reduction in the cell survival fraction. It can be considered a dose corresponding to a reduction of −*lnS* in cell survival although it is not an actual dose used.

Thus,

(3) $$BED=d\left(1+\frac{d}{\alpha /\beta }\right)$$

For a treatment in *n* fractions with a fractional dose of *d* and *a* total dose of *D*(= *nd*),

(4) $$BED=D\left(1+\frac{d}{\alpha /\beta }\right)$$

**Figure 2:**
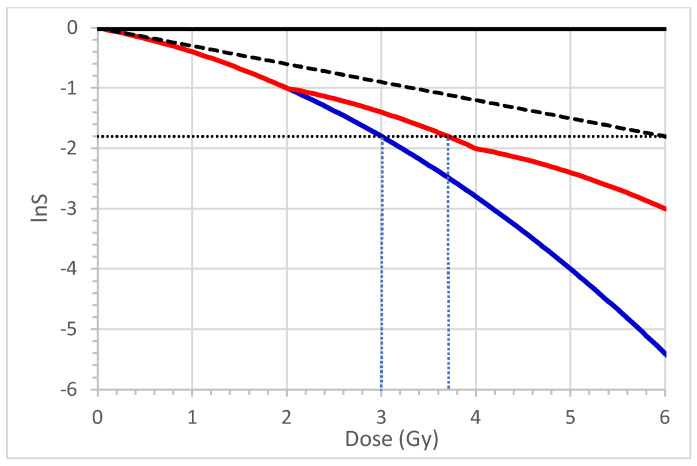
The linear-quadratic model. BED of 6 Gy (length of black line (top)), initial slope α of survival curve (black dashed line), 3-fraction survival curve (red line), single-fraction survival curve (blue line). α = 0.3 Gy, β = 0.1 Gy^2^. Horizontal dotted line indicates the lnS value of 1.8. The left vertical dotted line indicates the dose (3.0 Gy) corresponding to lnS = −1.8 for the blue curve, and the right vertical dotted line indicates the corresponding dose of 3.7 Gy for the red curve.

Figure 2 displays the cell survival fraction (lnS) as a function of the dose (D) described by the LQ equation. The blue curve represents a single fraction, the red curve includes three fractions, and the black dashed line is the tangent line with slope α. Corresponding to lnS = −1.8 and BED = 6 Gy (length of black line), the doses for the single-fraction and 3-fraction cell survival curves are 3.0 Gy and 3.7 Gy, as indicated by the two vertical dotted lines in Figure 2, respectively.

### 4.2. Biological Equivalent Dose and Conventional EQD_2_ Calculation

As the BED is a function of dose (*d* and *D*) and the number of fractions, different fractionations lead to different BED values for the same dose. For different fractionations with the same BED, the corresponding doses are considered as equivalent and called biological equivalent doses (EQDs). Suppose two treatment schemes have two different numbers of fractions, n_1_ and n_2_. Their BEDs are determined by

(5) $${BED}_{1}\left(n_{1},\;d_{1}\right)=n_{1}d_{1}\left(1+\frac{d_{1}}{\alpha /\beta }\right)$$

(6) $$D_{1}=n_{1}d_{1}$$

and

(7) $${BED}_{2}\left(n_{2},\;d_{2}\right)=n_{2}d_{2}\left(1+\frac{d_{2}}{\alpha /\beta }\right)$$

(8) $$D_{2}=n_{2}d_{2}$$

If two treatment schemes have the same BED, *D*_2_ is equivalent to *D*_1_, and vice versa, i.e.,

(9) $${BED}_{2}\left(n_{2},\;d_{2}\right)={BED}_{1}\left(n_{1},\;d_{1}\right)$$

In this case, either *BED*_2_ or *BED*_1_ can be simply expressed as the BED. Solving Equation (7) for *d*_2_, we obtain

(10) $$d_{2}=\frac{-n_{2}+\sqrt{{n_{2}}^{2}+4\left(\frac{n_{2}}{\alpha /\beta }\right)BED}}{2\left(\frac{n_{2}}{\alpha /\beta }\right)}$$

According to Equations (7) and (8), *D*_2_ can be expressed as

(11) $$D_{2}=\frac{BED}{\left(1+\frac{d_{2}}{\alpha /\beta }\right)}$$

As a result, *D*_1_ can be converted to *D*_2_, and vice versa. If d_2_ = 2 Gy, *D*_1_ is thus converted to the standard 2 Gy/fraction treatment. In this case, *D*_2_ can be labeled as *EQD*_2_, and

(12) $${EQD}_{2}=\frac{BED}{\left(1+\frac{2}{\alpha /\beta }\right)}$$

Since the fractional dose is 2 Gy and the total dose is *EQD*_2_ for the standard fractionation regimen, the number of fractions directly corresponding to *EQD*_2_, *n*_2_, is, by definition, the ratio of *EQD*_2_ and 2 Gy, assuming the fractional prescription dose is 2 Gy for the standard fractionation regimen

(13) $$n_{2}=\frac{{EQD}_{2}}{2Gy}$$

*EQD*_2_ can be used for both the target and normal tissues, but the number of fractions calculated using (13) for the normal tissue, *n_N_*, is usually different from that calculated for the target, *n_T_*, i.e.,

(14) $$n_{N}\neq n_{T}$$

because usually EQD_2_(N) is not equal to EQD_2_(T). Here, N stands for normal tissue and T for target or tumor.

### 4.3. Fractionation-Specific Equivalent Dose (FEQD)

Given a total dose *D* = *D*_1_, for fractionation regimen 1, the total dose D_2_ for fractionation regimen 2 is equivalent to D_1_ if they have the same BED value. *D*_2_ is thus the biological equivalent dose of *D*_1_. As *D*_2_ is also a function of the number of fractions, *n*_2_, it is thus called the fractionation-specific equivalent dose, labeled as *FEQD*(*n*_2_) in this study. Removing the subscript for all above parameters, we have

(15) $$FEQD\left(n\right)=nd$$

The equivalent fractional dose d can be obtained by Equation (10).

For the standard 2 Gy fractional dose regimen, we have

(16) $$FEQD\left(n\right)={FEQD}_{2}(n)$$

In this case, the target dose, d_T_, is 2 Gy, and *FEQD*_2_(*n_T_*) for the target is equal to the conventional *EQD*_2_(*T*). As the target and normal tissues are treated in the same fractionation regimen, *FEQD*_2_(*n_N_*) for the normal tissues and *FEQD*_2_(*n_T_*) for the target should have the same number of fractions, i.e.,

(17) $$n_{N}=n_{T}=n$$

while the equivalent fractional dose, *d_N_*, for the normal tissues may not be 2 Gy. The subscript “2” of *FEQD*_2_(*n*) for the normal tissues still refers to the target dose (*d_T_* = 2 Gy) not *d_N_*. *d_N_* can be determined by Equation (10). Thus, *FEQD*_2_(*n*) is not the EQD_2_ for normal tissues.

## 5. Conclusions

The EQD_2_ has limitations in describing biological equivalent dose for normal tissues since it does not match its definition and is not a realistic dose quantity. While the EQD_2_ can be used as an abstract quantity to compare different fractionation regimens in terms of biological effectiveness, it is not practical to use the EQD_2_ to calculate tolerance doses or dose constraints for various fractionation regimens. Instead, the FEQD(n) can be directly used to describe the normal tissue dose constraints for any fractionation regimens. In particular, the FEQD_2_(n) gives the correct fractional dose and number of fractions for the standard fractionation regimen. The FEQD(n) curves of the dose constraints derived for the brainstem, spinal cord, rectum, and lung, which correspond to the toxicity rates determined by the clinical outcomes, can be directly used in clinical practice.

## Figures and Tables

**Table 1:** Comparisons between different numbers of fractions.

Structure	BED (Gy)	D (Gy)	d (Gy)	n	α/β (Gy)
Target	72.00	60.00	2.00	30	10
	79.20	66.00	2.00	33	10
	84.00	70.00	2.00	35	10
Brainstem	D = D_max_				
	92.88	54.00	1.80	30	2.5
	89.35	54.00	1.64	33	2.5
	87.33	54.00	1.54	35	2.5
	D = EQD_2_				
	92.88	51.60	2.00	26	2.5
	89.35	49.64	2.00	25	2.5
	87.33	48.51	2.00	24	2.5

Note: BED is calculated for different fractionation regimens. D represents prescribed dose for target and maximum dose for the brainstem, d is the fractional dose, n is the number of fractions.

**Table 2:** Comparisons of maximum dose between FEQD_2_ and EQD_2_ for different BEDs.

BED (Gy)	D (Gy)	d (Gy)	n	α/β (Gy)	FEQD_2_/EQD_2_
92.88	D = FEQD_2_				
	54.00	1.80	30	2.5	1.047
	55.52	1.68	33	2.5	1.076
	56.46	1.61	35	2.5	1.094
	D = EQD_2_				
	51.60	2.00	26	2.5	
89.35	D = FEQD_2_				
	54.00	1.64	33	2.5	1.088
	54.90	1.57	35	2.5	1.106
	52.54	1.75	30	2.5	1.058
	D = EQD_2_				
	49.64	2.00	25	2.5	
87.33	D = FEQD_2_				
	54.00	1.54	35	2.5	1.113
	53.12	1.61	33	2.5	1.095
	51.69	1.72	30	2.5	1.066
	D = EQD_2_				
	48.51	2.00	24	2.5	

Note: BED is calculated for different fractionation regimens. D represents the maximum dose for the brainstem. d is the fractional dose, and n is the number of fractions.

**Table 4:** Comparisons between FEQD2 and EQD2 derived from real cases for the spinal cord.

Structure	Reference	BED (Gy)	D (Gy)	d (Gy)	n	α/β (Gy)	Toxicity Rate
Target	D = Prescribed dose						
	Marcus [25]	59.26	50.00	1.85	27	10	
	Wara [26]	79.43	58.07	3.66	25	10	
	D = FEQD_2_						
	Marcus	59.26	49.38	2.00	25	10	
	Wara	79.43	66.19	2.00	33	10	
Spinal cord	D = D_max_						
	Marcus	68.77	47.83	1.71	28	3.9	0.4%
	Wara	90.66	53.74	2.92	25	3.9	5.2%
	D = FEQD_2_						
	Marcus	68.77	46.41	1.88	25	3.9	0.4%
	Wara	99.76	61.11	1.89	33	3.9	5.2%
	D = EQD_2_						
	Marcus	68.77	45.46	2.00	23	3.9	0.4%
	Wara	90.66	59.93	2.00	30	3.9	5.2%

Note: D represents prescribed dose for target and maximum dose for the brainstem, d is the fractional dose, n is the number of fractions.

**Table 5:** Comparisons between FEQD_2_ and EQD_2_ derived from real cases for the rectum.

Structure	Reference	BED (Gy)	D (Gy)	d (Gy)	n	α/β (Gy)	Toxicity Rate
Target	D = Prescribed dose						
	Al-Mamgani [29]	182.00	78.00	2.00	39	1.5	
	Fonteynea [28]	178.89	76.67	2.02	38	1.5	
Rectum	D = D_mean_						
	Al-Mamgani	64.62	46.30	1.19	39	3	3.0%
	Fonteynea	79.43	54.00	1.41	38	3	7.5%
	D = FEQD_2_						
	Al-Mamgani	64.62	46.30	1.19	39	3	3.0%
	Fonteynea	79.43	54.00	1.41	38	3	7.5%
	D = EQD_2_						
	Al-Mamgani	64.62	38.77	2.00	19	3	3.0%
	Fonteynea	79.43	47.66	2.00	24	3	7.5%

Note: D represents prescribed dose for target and maximum dose for the brainstem, d is the fractional dose, n is the number of fractions.

**Table 6:** Comparisons between FEQD_2_ and EQD_2_ derived from real cases for the lung.

Structure	Reference	BED (Gy)	D (Gy)	d (Gy)	n	α/β (Gy)	Toxicity Rate
Target	D = Prescribed dose						
	Wang [30]	74.34	63.00	1.80	35	10	
	Baker [31]	100.00	50.00	10.00	5	10	
	D = FEQD_2_						
	Wang	74.34	61.95	2.00	31	10	
	Baker	100.00	83.30	2.00	42	10	
Lung	D = D_mean_						
	Wang	19.09	16.50	0.47	35	3.0	13.5%
	Baker	7.17	5.3	1.06	5	3.0	1.6%
	D = FEQD_2_						
	Wang	19.09	16.30	0.52	31	3.0	13.5%
	Baker	7.17	6.80	0.16	42	3.0	1.6%
	D = EQD_2_						
	Wang	19.09	11.50	2.00	6	3.0	13.5%
	Baker	7.17	4.30	2.00	2	3.0	1.6%

Note: D represents prescribed dose for target and maximum dose for the brainstem, d is the fractional dose, n is the number of fractions.

## Data Availability

The data presented in this study are available.
